# De Novo Design of AC-P19M, a Novel Anticancer Peptide with Apoptotic Effects on Lung Cancer Cells and Anti-Angiogenic Activity

**DOI:** 10.3390/ijms232415594

**Published:** 2022-12-09

**Authors:** Min Kyoung Shin, Bo-Young Jang, Kyung-Bin Bu, Seung-Ho Lee, Dong-Hee Han, Jin Wook Oh, Jung-Suk Sung

**Affiliations:** Department of Life Science, Dongguk University-Seoul, Goyang 10326, Republic of Korea

**Keywords:** anticancer peptide (ACP), in silico analysis, cell lysis, apoptosis, EMT inhibition, anti-angiogenic

## Abstract

Despite the current developments in cancer therapeutics, efforts to excavate new anticancer agents continue rigorously due to obstacles, such as side effects and drug resistance. Anticancer peptides (ACPs) can be utilized to treat cancer because of their effectiveness on a variety of molecular targets, along with high selectivity and specificity for cancer cells. In the present study, a novel ACP was de novo designed using in silico methods, and its functionality and molecular mechanisms of action were explored. AC-P19M was discovered through functional prediction and sequence modification based on peptide sequences currently available in the database. The peptide exhibited anticancer activity against lung cancer cells, A549 and H460, by disrupting cellular membranes and inducing apoptosis while showing low toxicity towards normal and red blood cells. In addition, the peptide inhibited the migration and invasion of lung cancer cells and reversed epithelial-mesenchymal transition. Moreover, AC-P19M showed anti-angiogenic activity through the inhibition of vascular endothelial growth factor receptor 2 signaling. Our findings suggest that AC-P19M is a novel ACP that directly or indirectly targets cancer cells, demonstrating the potential development of an anticancer agent and providing insights into the discovery of functional substances based on an in silico approach.

## 1. Introduction

Cancer is caused by genetic and epigenetic alterations leading to dysregulated cell growth. Cancerous cells can be distinguished from normal cells by rapid proliferation, the evasion of cell cycle arrest and apoptotic signals, and the invasion and metastasis to other tissues [1]. As a leading cause of death worldwide, cancer poses a substantial burden to public health and economy, especially with the growing aging population [2]. Several options are available for cancer treatment, including chemotherapy, radiation therapy, surgery, targeted cell therapy, and immunotherapy [3]. Both the conventional and newly developed methods, however, are often accompanied by side effects, drug resistance, and reduced efficacy. Therefore, efforts are continuously being made to discover new substances with anticancer properties [4].

Among the factors that influence the development and progression of cancer, epithelial-mesenchymal transition (EMT) is known to be crucial in various cancer types. EMT is a process in which cells lose their adhesion and polarity while acquiring the characteristics of mesenchymal stem cells. During the process of EMT, the mobility and plasticity of cancer cells and the heterogenicity within the tumor increase, causing drug resistance and loss of sensitivity [5,6].

In addition, various molecules are secreted in the tumor microenvironment (TME), which act as autocrine and paracrine signals to create pro-tumor conditions [7]. Growth factors such as the vascular endothelial growth factor (VEGF) are upregulated and secreted steadily during cancer initiation and progression, which in turn promotes tumor growth and angiogenesis [8]. The newly formed blood vessels help the growth and maintenance of cancer cells by efficiently supplying nutrients and oxygen to the cancer cells. Cancer metastasis is induced when the malignancy of cancer cells and vascularization in tumor tissue are enhanced, which are characteristics associated with aggressive forms of cancer and with poor prognosis [9,10]. Therefore, EMT and angiogenesis serve as major targets for cancer treatment, and their suppression increases the survival rate of patients.

Peptides are short amino acid (AA) chains that possess various physiological activities, such as anticancer, antibacterial, and immunomodulatory activity. Peptides have a wide range of applications as they show high diversity and specificity, molecular flexibility, and biocompatibility, while exerting relatively low toxicity compared to other small molecules [11]. Among them, anticancer peptides (ACPs) are drawing much attention in the development of new anticancer agents, since they show selective effects against cancer cells owing to their high net charge and amphipathicity [4,12]. There are several ways to discover bioactive peptides; natural peptides in living organisms can be identified, and new peptides can be designed de novo. Analyzing the structure and physiological properties of peptides has become feasible, and with the development of artificial intelligence (AI) technology, tools for predicting and improving the functionality of peptides have been devised, leading to the generation of novel functional peptides based on accumulated biological data [13,14,15].

In this study, we implemented an in silico method to identify peptides with anticancer activity and to investigate their molecular mechanisms. A novel ACP, AC-P19M, was de novo designed by utilizing various in silico tools for AI-based functional prediction and physiochemical property calculation, as well as applying biological knowledge related to functionality. The peptide showed cytotoxicity against lung cancer cells A549 and H460, while showing low toxicity against normal lung cells and red blood cells. AC-P19M was found to induce apoptosis by disrupting the cell membrane of cancer cells. In addition, AC-P19M inhibited cell migration and invasion as well as EMT markers of cancer cells. Finally, the peptide exerted anti-angiogenic activity on endothelial cells by attenuating VEGF signaling. Taken together, the results demonstrated that AC-P19M is a novel ACP that targets cancer cells both directly and indirectly, serving as a potential anticancer agent for therapeutic applications.

## 2. Results

### 2.1. Design of a Novel Anticancer Peptide and Its Functional Validation

**Figure 1:**
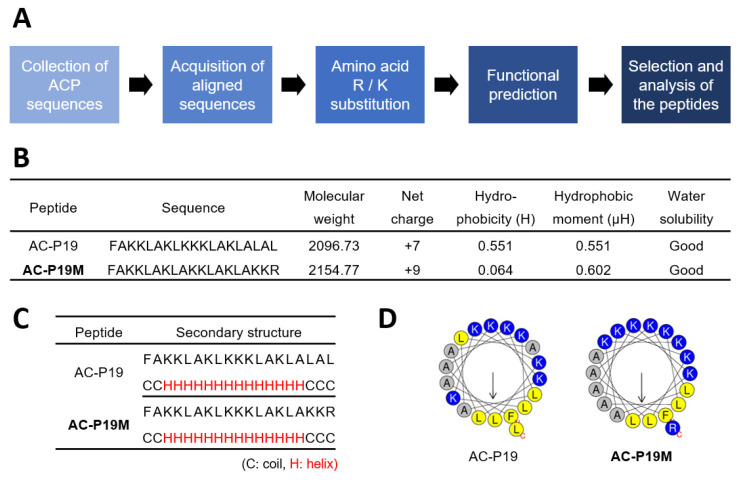
De novo design and characterization of ACPs. (**A**) Scheme of de novo design of novel ACPs. (**B**) Physicochemical properties of AC-P19, aligned sequence from 19-mer ACPs, and AC-P19M, modified sequence of AC-P19 with arginine (R) and lysine (K) substitutions. The peptide sequence, molecular weight, net charge, hydrophobicity, hydrophobic moment, and water solubility of AC-P19 and AC-P19M were calculated. (**C**) The secondary structures of the two peptides were predicted to be helical. (**D**) Amino acid configurations of the helix structures of the peptides are presented.

In order to design a novel ACP, we implemented a rational strategy based on in silico methods (Figure 1A). First, peptide sequences with experimentally validated anticancer activity were collected from the CancerPPD database. Peptides of lengths ranging from 15 to 20 AAs were used in consideration of the synthesis and cost, and a total of six peptide sequences were produced by WebLogo for each length (Supplementary Table S1). The AA composition of a peptide is strongly correlated with its function. ACPs are rich in cationic and hydrophobic residues, such as arginine (R) and lysine (K), and exert cytotoxicity on cancer cells. Therefore, we substituted the individual AAs of aligned sequences with Ks and Rs to enhance their anticancer potential, generating a series of candidate peptides. These were then subjected to functional prediction using AI-based web tools for anticancer, cell penetration, and hemolysis activity (Supplementary Table S2). The peptide sequences were examined for anticancer potency along with high cell penetration and low hemolytic activity. AC-P19, a sequence derived from 19-mer ACPs, and AC-P19M, the most potent sequence developed based on AC-P19, were finally selected.

AC-P19M was designed by substituting K and R for the last three AAs at the C-terminal of AC-P19. In terms of physiochemical properties, the sequence modification resulted in a net charge increase from +7 to +9 and a dramatic decrease in hydrophobicity, while the hydrophobic moment was increased (Figure 1B). The secondary structure was predicted using PEP2D and visualized by UCSF Chimera, and both peptides were predicted to have helical structures (Figure 1C and Supplementary Figure S1). When the distribution of AAs in the helix was observed using Heliquest, it was found that the hydrophilic and hydrophobic faces were distinctly separated in AC-P19M, in line with the increase in hydrophobic moment that is indicative of molecular amphipathicity (Figure 1D). As AC-P19 and AC-P19M were characterized via in silico analysis, further experimental verification was conducted.

### 2.2. Anticancer Activity of the Designed Peptides AC-P19 and AC-P19M

**Figure 2:**
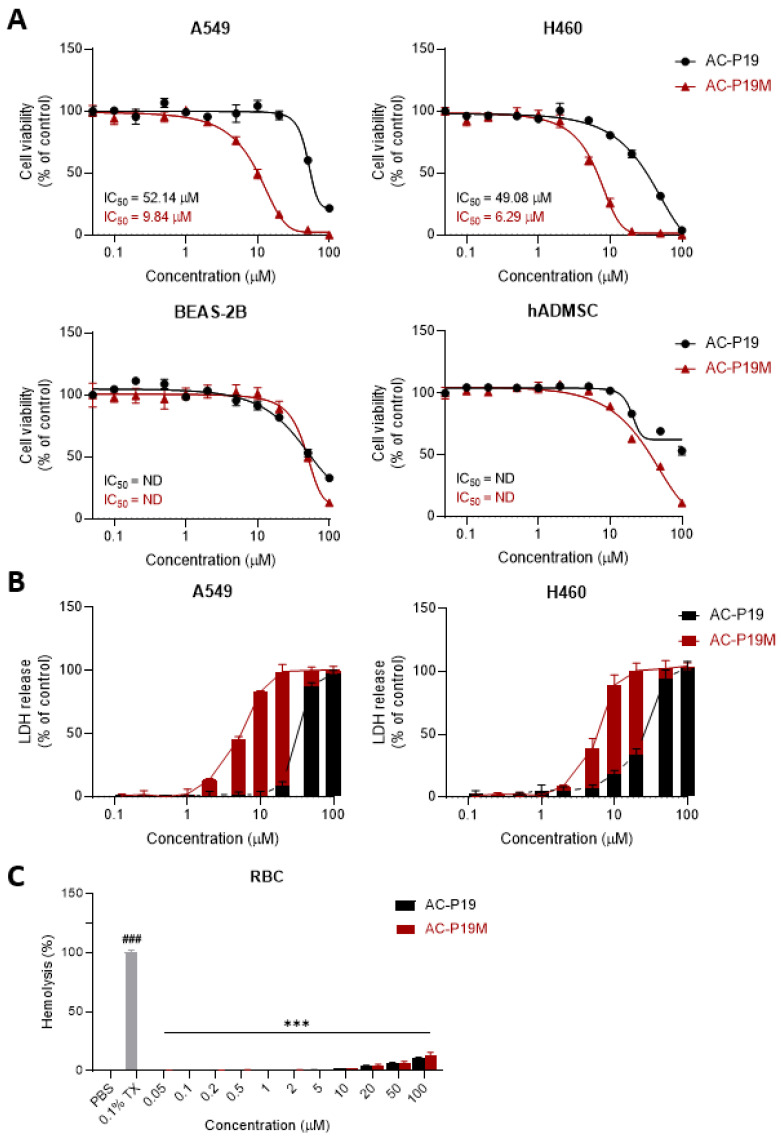
Anticancer activities of the peptides AC-P19 and AC-P19M. (**A**) The relative cell viability of lung cancer cells and normal cells was examined after AC-P19 and AC-P19M treatments at concentrations ranging from 0.1 μM to 100 μM. Lung cancer cells were more susceptible to peptide treatment than normal cells, and the decrease in cancer cell viability was higher in the AC-P19M-treated group. (**B**) The cytotoxic effects of the peptides on lung cancer cells were confirmed through an LDH release assay. The analyses were conducted for a range of peptide concentrations between 0.1 μM and 100 μM. LDH release at levels comparable to the positive control (0.1% Triton X-100; 0.1% TX) was obtained with a lower concentration of AC-P19M compared with AC-P19. (**C**) For both peptides (up to 100 μM), hemolysis was observed to be lower than 15% of that observed upon treatment with 0.1% TX. Data are presented as the mean ± SEM. ###, *p* < 0.001 compared to the control group; ***, *p* < 0.001 compared to the 0.1% TX-treated group.

To confirm the anticancer activity of the newly designed peptides AC-P19 and AC-P19M, they were tested on human cell lines. A cell viability assay was conducted by treating lung cancer cells A549 and H460, normal lung cell BEAS-2B, and primary adipose-derived mesenchymal stem cells (hADMSCs) with various concentrations of the two peptides. A dose-response curve was constructed for each cell line, and the half-maximum inhibitory concentrations (IC_50_) of the peptides were calculated. As shown in Figure 2A, the viability of cancer cells decreased upon peptide treatment. Specifically, the IC_50_ of AC-P19M was at least five times lower than that of AC-P19, indicating much stronger inhibitory activity. Despite testing with peptide concentrations of up to 100 μM, we were not able to reach the IC_50_ in normal or primary cells, demonstrating that the designed peptides exhibited cancer-cell-specific cytotoxicity. Moreover, the modification of AC-P19 into AC-P19M improved the anticancer effect without increasing toxicity to normal cells.

As numerous ACPs induce cell lysis, the effect of AC-P19 and AC-P19M on the cancer cell membrane was then observed. After the treatment of A549 and H460 cells with AC-P19 and AC-P19M for 6 h, the culture supernatant was analyzed for released lactate dehydrogenase (LDH), which is an indicator of loss of cell membrane integrity. Both peptides induced LDH release at levels comparable to those observed in positive controls treated with 0.1% Triton X-100 at concentrations up to 100 μM, where AC-P19M induced the maximum release of LDH at concentrations above 20 μM in cancer cells. These results indicated that the peptides disrupt cell membranes, leading to cell death.

In addition to the cytotoxicity test, a hemolysis assay was performed to evaluate the toxicity of the peptides. PBS was used as a negative control causing no hemolysis, and 0.1% Triton X-100 was used as a positive control with 100% hemolytic activity. Peptide treatment induced the hemolysis of human red blood cells by 15% or less at the highest concentration compared with the positive control. Although AC-P19M showed slightly higher hemolytic activity than AC-P19, the difference was not comparable with the anticancer potency of the peptides. Collectively, AC-P19M, which underwent sequence modification, exhibited stronger anticancer function, as well as biocompatibility. Therefore, further investigation was conducted to explore the mechanisms underlying the anticancer potential of the peptides.

### 2.3. Induction of Apoptosis in Lung Adenocarcinoma Cells by AC-P19M

**Figure 3:**
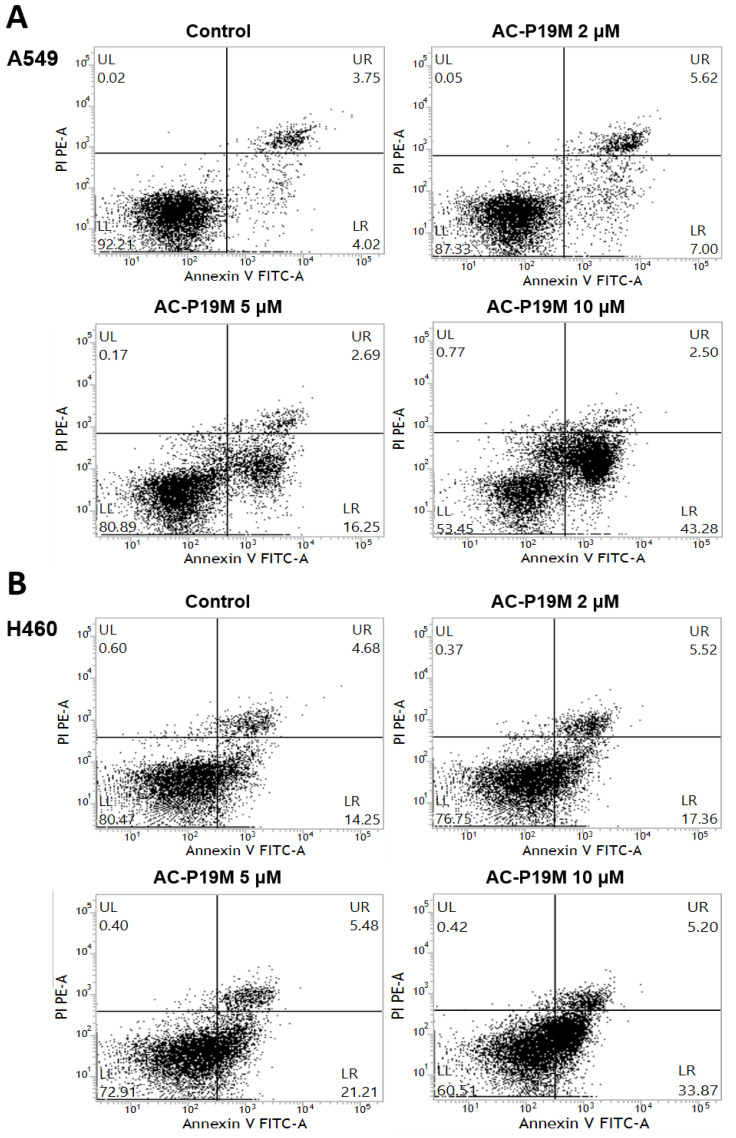
Apoptosis induction by AC-P19M in lung cancer cells. Apoptosis in (**A**) A549 and (**B**) H460 cells upon AC-P19M treatment was evaluated by flow cytometry analysis through Annexin V-FITC and PI double staining (UL: upper left, UR: upper right, LL: lower left, LR: lower right). The analysis was performed by comparing the control (PBS-treated) and the peptide treatment groups (2, 5, 10 μM of AC-P19M). The numbers of both early (Annexin V-FITC+ single positive) and late (Annexin V-FITC/PI double positive) apoptotic cells increased with increasing peptide concentration. The representative images of duplicate samples for the apoptosis assay were shown.

Since the anticancer function of AC-P19M is related to cell membrane disruption and cellular damage, we investigated whether the peptide could induce apoptosis in lung cancer cells by flow cytometry. Peptide concentrations of 2, 5, and 10 μM were treated, by considering the IC_50_ values of the two cancer cells. Apoptosis was analyzed by double staining the cells with Annexin V and propidium iodide (PI). FITC-conjugated Annexin V can detect phosphatidylserine exposed to the outside of the cellular membrane in apoptotic cells, whereas PI, a cell impermeant dye, stains cells with damaged membranes. Both the early (Annexin V-FITC+/PI-) and late (Annexin V-FITC+/PI+) apoptotic cell populations enlarged with increasing peptide concentration (Figure 3). Additional cell cycle analysis showed no significant effect of peptide treatment on the cell cycle (Supplementary Figure S2).

**Figure 4:**
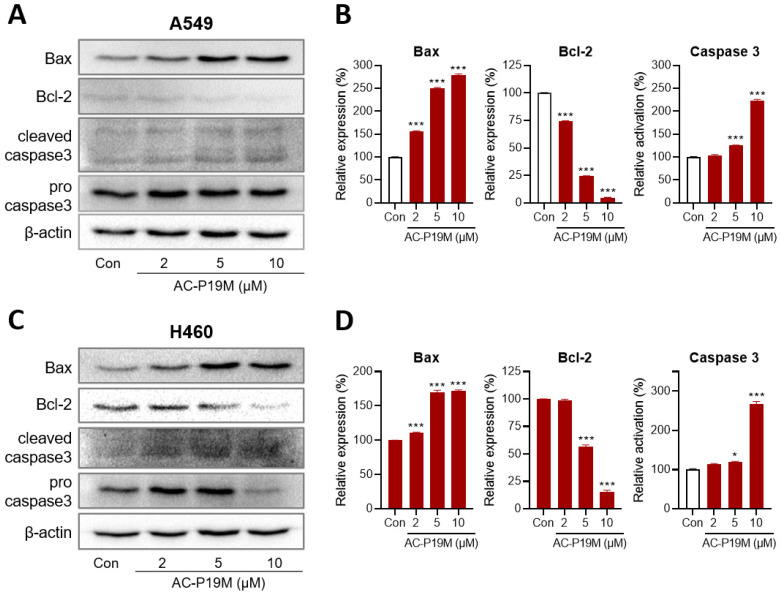
Effect of AC-P19M on the expression of apoptosis markers in lung cancer cells. Western blot analysis was conducted to measure the protein levels of apoptosis markers, Bax, Bcl-2, and caspase 3. AC-P19M treatment was performed on (**A**,**B**) A549 and (**C**,**D**) H460 cells for 24 h. Quantitative analysis of the western blot showed that the protein levels of Bax and caspase 3 increased, while those of Bcl-2 decreased in a dose-dependent manner after AC-P19M treatment. Data are presented as the mean ± SEM. *, *p* < 0.05; ***, *p* < 0.001 compared to the control group.

The changes in the expression levels of cell death markers were measured to confirm the induction of apoptosis by AC-P19M. Apoptosis, a distinctive form of programmed cell death, is proceeded by the consecutive regulation and activation of effector proteins. Under cellular stress or death signals, the expression of the pro-survival molecule Bcl-2 is suppressed; whereas, that of the pro-apoptotic molecule Bax is upregulated. In the late phase of apoptosis, caspase 3, an executioner enzyme, is activated upon cleavage, ultimately leading to irreversible cell death that involves DNA and protein degradation. Thus, the protein levels of Bax, Bcl-2, and caspase 3 were measured by western blot analysis (Figure 4). Bax protein levels were upregulated up to 2.5-fold in cells treated with the peptide in a concentration-dependent manner. Similarly, Bcl-2 levels were significantly decreased, showing almost no expression in cells treated with 10 μM of AC-P19M. In the case of caspase 3, a more than 2-fold increase was observed upon peptide treatment. These results demonstrated that AC-P19M is an ACP that induces apoptosis through the Bcl-2/Bax/caspase 3 cascade.

### 2.4. Inhibition of Cell Migration and Invasion by AC-P19M

The enhancement of cell migration and invasion is involved in tumor progression and metastasis, for which EMT is responsible in various types of cancer. Since the process is frequently induced during the progression of cancer and as the cancer becomes more aggressive, EMT inhibition is often used to control cancer development with the simultaneous elimination of cancer cells. As peptides can target a variety of intracellular and cell surface molecules, we investigated whether AC-P19M can affect EMT in lung cancer cells, in addition to directly destroying cancer cells.

**Figure 5:**
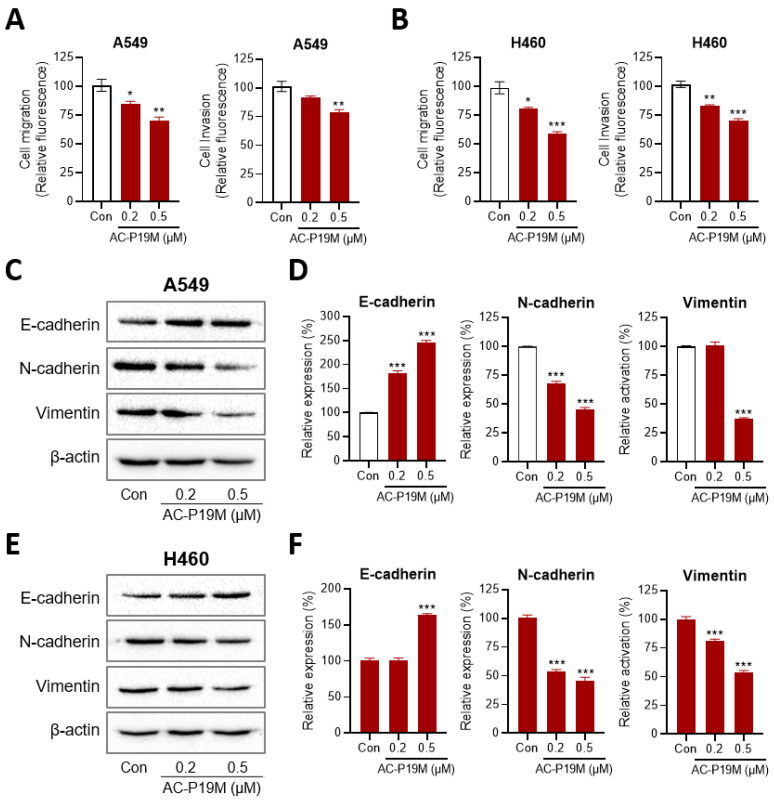
Inhibition of EMT by AC-P19M in lung cancer cells. Lung cancer cells were treated with 0.2 μM or 0.5 μM of AC-P19M to perform cell migration and invasion assays. The migratory and invasion activity in both (**A**) A549 and (**B**) H460 cells decreased after peptide treatment. (**C**,**E**) Western blot analysis showed changes in protein levels of EMT markers upon AC-P19M treatment in (**C**,**D**) A549 and (**E**,**F**) H460 cells. The protein levels of the epithelial cell marker, E-cadherin, increased, whereas those of the mesenchymal cell markers, N-cadherin and Vimentin, increased upon peptide treatment in a dose-dependent manner. Data are presented as the mean ± SEM. *, *p* < 0.05; **, *p* < 0.01; ***, *p* < 0.001 compared to the control group.

First, the effect of AC-P19M treatment on cell migration and invasion was confirmed based on Transwell assays. Cells were treated with 0.2 or 0.5 μM AC-P19M, at concentrations that did not affect cell viability. The degree of cell movement across a Transwell or through basement membrane extract (BME)-coated insert was measured to assess cell migration and invasion. As a result, AC-P19M inhibited cell migration and invasion by more than 20% in both A549 and H460 cells (Figure 5A,B). Subsequently, the change in the expression of EMT-related proteins was investigated after the peptide treatment. AC-P19M increased the expression levels of E-cadherin, an epithelial marker, while decreasing the expression levels of N-cadherin and vimentin, the mesenchymal markers (Figure 5C–F). The results confirmed that AC-P19M could reverse the mesenchymal properties of cancer cells, inhibiting EMT.

### 2.5. Anti-Angiogenic Activity of AC-P19M on HUVECs

As the high proliferation rates of cancer cells increase oxygen and nutrient requirements, tumor progression is heavily accompanied by blood vessel formation and vascularization. Angiogenesis is not only crucial for the survival and growth of cancer cells in the primary site, but it also allows the cancer cells to metastasize into new sites. In the tumor microenvironment, the secretion of soluble factors, such as growth factors and cytokines, is increased, which affects the surrounding stromal cells. In particular, the increased secretion of VEGF by cancer cells promotes the angiogenesis of endothelial cells. Accordingly, the effect of AC-P19M on angiogenesis was explored using human umbilical vein endothelial cells (HUVECs).

**Figure 6:**
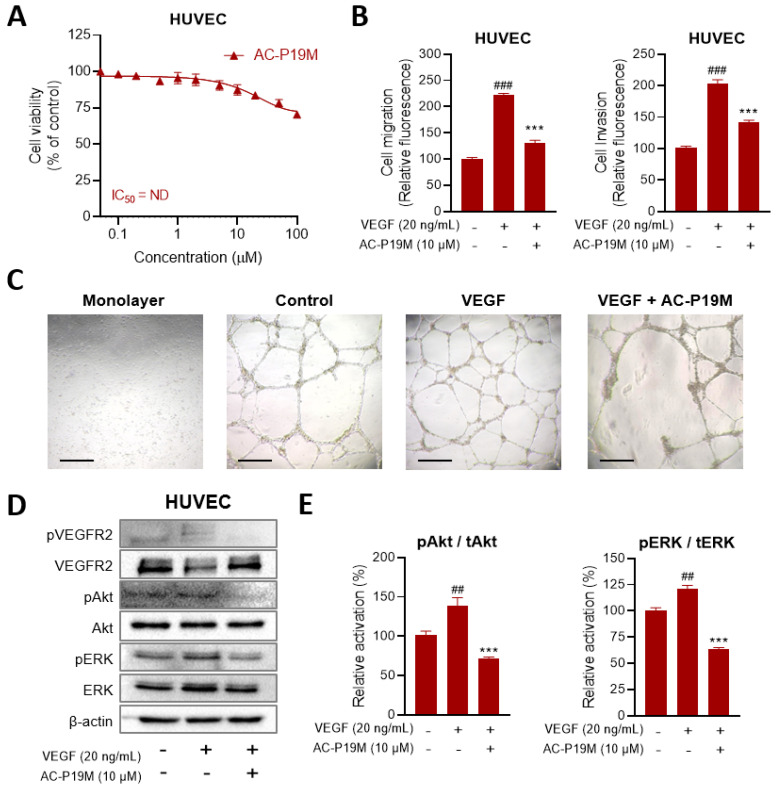
Anti-angiogenic activity of AC-P19M on HUVECs. (**A**) The relative cell viability of HUVECs upon AC-P19M treatment was confirmed in the concentration range of 0.1 μM to 100 μM. The peptide had no significant effect; the viability was maintained at more than 70% after peptide treatment at the maximum concentration. (**B**) The effects of VEGF and AC-P19M on cell migration and invasion were evaluated. VEGF increased the migration and invasion of HUVECs, but cotreatment with AC-P19M attenuated this effect. (**C**) The effect of AC-P19M on angiogenesis of HUVECs was observed by performing a tube formation assay. The absence of Matrigel led to the formation of a monolayer of HUVECs without tube formation. When the cells were incubated with 20 ng/mL of VEGF, the formation of the capillary structure was enhanced, along with the increased network and tube length. However, cotreatment with VEGF and AC-P19M resulted in the disruption of tube structure and branching with the aggregation of cells, demonstrating the anti-angiogenic activity of AC-P19M. The scale bar represents 100 μm. (**D**,**E**) The activation of VEGFR2 signaling in HUVECs was measured by western blot analysis. Cotreatment of cells with VEGF and AC-P19M almost completely suppressed VEGFR2 phosphorylation compared with the VEGF treatment. Moreover, the downstream of the VEGFR2-signaling pathway was affected, where the activations of Akt and ERK were attenuated by AC-P19M treatment. Data are presented as the mean ± SEM. ##, *p* < 0.01; ###, *p* < 0.001 compared to the control group; ***, *p* < 0.001 compared to the VEGF-only treated group.

First, the cell viability of HUVECs following AC-P19M treatment was determined. The cells were less sensitive to the peptide when compared with other normal cells, showing cell viability above 70% upon the treatment with up to 100 μM of peptide (Figure 6A). A concentration of 10 μM was selected for subsequent analysis in consideration of the anticancer activity of AC-P19M against lung cancer cells. For blood vessels to be newly formed, it is important for endothelial cells to migrate near to the tumor. Therefore, VEGF or VEGF and peptides were cotreated when seeding HUVECs in the top chamber of the Transwell to observe the effect on cell migration and invasion. As shown in Figure 6B, the cell migration and invasion, that were increased by VEGF, decreased upon AC-P19M treatment. Subsequently, a tube formation assay using Matrigel was performed to evaluate the anti-angiogenic effect of AC-P19M. On top of the solidified growth-factor-reduced Matrigel, HUVECs diluted in the endothelial basal medium were seeded and served as the control, compared with the single treatment of 20 ng/mL VEGF or cotreatment with 10 μM AC-P19M. HUVECs seeded without Matrigel were set as the negative control, which resulted in a monolayer of cells without tube formation. It was confirmed that angiogenesis was promoted by increased tube length and branching by VEGF compared to the control (Figure 6C). When AC-P19M was cotreated with VEGF, the number of branches and tubes were significantly decreased. These results suggested that AC-P19M exhibits an anti-angiogenic effect by contributing to the reduction of tubular morphology.

As VEGF-induced VEGF receptor 2 (VEGFR2) signaling plays an important role in the angiogenesis of endothelial cells, the effect of the peptide on the VEGFR2 pathway was investigated. When VEGFR2 is activated, Akt and extracellular signal-regulated kinase 1/2 (ERK) are phosphorylated and induce cell survival and angiogenesis. In the AC-P19M treatment group, phosphorylation of VEGFR2 was almost completely inhibited compared with the VEGF treatment group, and the activations of Akt and ERK were also attenuated (Figure 6D, E). Based on these results, AC-P19M was found to have anti-angiogenic activity while inhibiting VEGF-VEGFR2/ERK/Akt signaling.

## 3. Discussion

Despite the endless ongoing research on cancer treatments, the fight against cancer has yet to end due to the complex biology of the disease. For the development of anticancer drugs, new bioactive molecules must be developed along with the elucidation of their molecular mechanisms of action [16]. ACPs serve as potential candidates of anticancer agents due to their high selectivity and therapeutic efficacy for cancer cells, and relatively low toxicity [17]. In this study, we identified a novel ACP that can simultaneously target the cancer cells and the tumor microenvironment to further enhance the anticancer effect. To this end, a rational de novo peptide design was performed by applying in silico methods such as various machine learning-based predictions and physiological calculations, and the anticancer effect of AC-P19M and the underlying mechanisms of its action were explored [18].

Cancer and normal cells differ in cell membrane composition, allowing ACPs to have increased selectivity for cancer cells. For example, negatively charged phosphatidylserines, which are phospholipids mainly distributed in the inner leaflet in normal cells, are rich in the outer leaflet of cancer cells [19]. Thus, ACPs, which form amphiphilic helical structures with a high net charge, can bind to the membranes of cancer cells or penetrate into the intracellular region. Accordingly, the most well-known mechanism of ACP action is the destruction of cancer cells by disrupting the cell membrane [20]. In this study, AC-P19, derived from the alignment of known 19-mer ACPs, initially had a high proportion of R and K compared with other candidate sequences, which resulted in a high positive charge and hydrophobic moment. The overall score of anticancer and cell-penetrating activities of AC-P19 exceeded those of other candidate peptides. AC-P19M disrupted cell membranes of lung cancer cells with a low IC_50,_ and exhibited high cancer cell selectivity with low toxicity against normal cells and erythrocytes. The substitution of three C-terminus AAs with R and K to generate AC-P19M enhanced the amphipathicity of the helical structure and improved anticancer function through increased interaction with the cancer cell.

The positive charge and helical structure of AC-P19M are also characteristics of antibacterial peptides (AMPs), which kill bacteria through their interaction with the negatively charged bacterial membrane [21]. Indeed, additional antibacterial experiments showed that AC-P19 and AC-P19M destroy bacteria with low minimum inhibitory and bactericidal concentrations in *Escherichia coli*, *Pseudomonas aeruginosa*, and *Bacillus subtilis* (Supplementary Table S3 and Figure S3). Patients who undergo chemotherapy or surgery have a higher risk of microbial infection, which can pose fatal consequences and reduce the efficiency of cancer treatment [22]. Since AC-P19M is found to have antibacterial effects in addition to anticancer activity, it has a high potential for various applications in cancer therapy.

Tumor invasion and metastasis are key features of highly lethal and aggressive cancers, making it difficult to cure cancer completely. In this regard, the EMT is a representative and major process that allows the invasion and metastasis of these malignant tumors [23]. During EMT, cells exhibit a decrease in the levels of cell adhesion molecules and an increase in cellular metabolism, thereby transitioning from an epithelial to a mesenchymal cell type. Mesenchymal cells, which are migratory and invasive, are critical drivers of tumor metastasis. The EMT-directed phenotypic changes are also responsible for the heterogenicity of tumor tissue, where individual cells undergo an unsynchronized transition that leads to changes, including those in cell proliferation, survival, senescence, immune response, and drug resistance [24]. An increase in the cell adhesion factor E-cadherin and a decrease in the mesenchymal stem cell markers N-cadherin and Vimentin in lung cancer cells indicated that AC-P19M reversed the EMT process. AC-P19M reduced the mobility and mesenchymal plasticity of cancer cells, suggesting that AC-P19M can be used as an anticancer agent that inhibits the growth of cancer cells as well as metastasis, which is currently a major challenge in treating cancer.

Tumor cells require the generation of new blood vessels to receive sufficient nutrients and oxygen to proliferate and survive [25]. As a result, cancer cells consume even the nutrients that the surrounding normal cells should normally receive, which leads to the starvation and death of otherwise healthy cells. Angiogenesis is rapidly induced by growth factors secreted from TME, supporting tumor growth and metastasis [26]. In particular, as the vascular system within the lungs is complex, the risk of metastasis and the difficulty of treatment are high during the process of EMT and neoangiogenesis in lung cancer. Among the angiogenic factors, VEGF not only induce the proliferation of endothelial cells but also increase the membrane permeability of blood vessels [27]. Since angiogenesis is mainly induced by phosphorylation of VEGFR2, it is pivotal to inhibit the activation of the VEGFR2 signaling, which can stimulate downstream molecules, such as Akt and ERK, when treating a malignant tumor [28]. AC-P19M treatment attenuated angiogenesis induced by VEGF and inhibited VEGFR2 activation, while exhibiting no significant cytotoxicity on HUVECs. Therefore, by confirming the anticancer activity as well as the anti-angiogenic activity of AC-P19M, our findings confirmed that AC-P19M could be effective in treating lung cancer.

As the understanding of cancer biology increases, the discovery of new anticancer targets and anticancer agents is becoming more feasible. In particular, owing to the advances in computational techniques, it is also possible to repurpose existing drugs and de novo design candidates through in silico screening, prediction, and modeling. Beyond discovering anticancer substances, a more effective strategy can be suggested by combining newly identified bioactive molecules with conventional drugs, standard chemotherapy, radiation therapy, and surgical excision [29]. AC-P19M discovered in this study is a novel multi-target ACP that not only directly destroys cancer cells but also induces apoptosis, inhibits EMT, and inhibits angiogenesis, and may be expected to exhibit synergistic effects when combined with other methods. These results demonstrated a new in silico approach for discovering anticancer peptides, providing insights into the design of functional peptides on different molecular targets with broadened functionalities.

## 4. Materials and Methods

### 4.1. Design and Characterization of ACP

In order to design novel ACPs, known ACP sequences of lengths ranging from 15 to 20 AAs were downloaded from the CancerPPD [30] database. The template sequence for each length was aligned from the collected sequences by WebLogo [31]. Individual AAs in the template sequences were randomly substituted with Ks and Rs, with a maximum substitution of five residues, to generated candidate peptides. Using AI-based web tools, the peptides were predicted to have anticancer (mACPpred [32], ACPred [33], ENNACT [34]), cell-penetrating (CellPPD [35], BchemRF-CPPred [36], MLCPP [37]), and hemolytic (HAPPENN [38], DBAASP [39] erythrocyte, HemoPI [40]) activities. The most potent peptide sequence was selected based on the overall scoring on the functional prediction.

A set of the aligned and modified sequences were subjected to the in silico analysis of physiochemical properties. The molecular weight, net charge, and water solubility were calculated using the Protein Calculator (https://pepcalc.com/protein-calculator.php accessed on 25 October 2022). Heliquest was used to analyze hydrophobicity, the hydrophobic moment, and the helical configuration of the peptides [41]. Finally, the secondary structure was predicted by the PEP2D tool, and the three-dimensional modeling of the peptides was conducted using UCSF Chimera [42,43].

### 4.2. Cell Lines and Culture Conditions

The human non-small cell lung cancer cell lines (A549 and H460), human normal bronchial epithelial cells (BEAS-2B), and HUVECs were purchased from American Type Culture Collection (ATCC, Manassas, VA, USA). A549, H460, and BEAS-2B cells were cultured in Roswell Park Memorial Institute Medium (RPMI 1640, Welgene, Daegu, Republic of Korea) containing 10% fetal bovine serum (FBS, Gibco, NY, USA) and 1% penicillin/streptomycin (Welgene). HUVECs were cultured in EGM™-2 Endothelial Cell Growth Medium-2 BulletKit™ (Lonza, Basel, Switzerland) with 1% penicillin/streptomycin (Gibco). The human adipose-derived mesenchymal cells (hADMSCs) were purchased from CEFO (Seoul, Republic of Korea) and cultured in CEFOgro™ Human MSC Growth Medium (CEFO). The cells were cultured in a humidified incubator with 5% CO_2_ at 37 °C.

### 4.3. Cell Viability and Cytotoxicity Test

For the cell viability test, the cells were seeded on 96-well plates at a density of 1 × 10^5^ cells/mL and incubated for 24 h. Peptide treatments at different concentrations were performed for 24 h to evaluate their effects on the cell viability. Quanti-Max WST-8 Cell Viability assay solution (Biomax, Seoul, Republic of Korea) was added to each well, and then the plate was incubated for 1 h. In order to measure the cytotoxicity of the peptides, the LDH release assay was performed using a Lactate Assay kit II (Sigma-Aldrich, St. Louis, MO, USA) according to the manufacturer’s instructions. Briefly, the cells were seeded on 96-well plates at a density of 1 × 10^5^ cells/mL and incubated for 24 h, followed by peptide treatment for 24 h. Then, 50 μL of lactate assay buffer was added to each well, and the cells were incubated for 15 min at room temperature. The absorbance was read at 450 nm using a Sunrise™ Absorbance microplate reader (TECAN, Männedorf, Switzerland), and the relative cell viability or cytotoxicity was calculated.

### 4.4. Apoptosis Assay and Cell Cycle Analysis

A549 and H460 cells were seeded on 60 mm dishes with a density of 5 × 10^5^ cells/mL and incubated for 24 h. Peptide treatments (2, 5, and 10 μM) were performed for 24 h. Cells in the supernatant and cells attached to the dish were collected in a 50 mL conical tube, and cell pellets were harvested through centrifugation at 4 °C and 1500 RPM for 5 min. Cell pellets were washed with 1 mL PBS and collected in a 1.7-mL tube. After removing the supernatant, cell pellets were resuspended in 1x binding buffer. Cells were diluted to reach a density of 1 × 10^6^ cells/mL and were stained with FITC-Annexin V apoptosis Detection Kit I (BD Biosciences, Franklin Lakes, NJ, USA). In each sample, 5 μL of Annexin V-FITC and 3 μL of PI were added, and the samples were incubated in the dark at room temperature for 15 min. Stained cells were immediately analyzed with a flow cytometer (BD Biosciences).

For cell cycle analysis, cells were prepared as described above for the apoptosis assay. After the cell density was adjusted, 300 μL of cell suspension was fixed by adding 700 μL of 100% ice-cold ethanol dropwise, which was incubated at 4 °C overnight. Cells were washed with PBS and resuspended with a cell cycle buffer containing 50 μg/mL PI (Sigma-Aldrich) and Rnase (Sigma-Aldrich). Cells were incubated at room temperature for 30 min and analyzed immediately using a flow cytometer (BD Biosciences).

### 4.5. Western Blotting

The cells were lysed using RIPA buffer (Biosolution, Seoul, Republic of Korea) containing protease and phosphatase inhibitor cocktails (Sigma-Aldrich). For the quantification of protein concentration, a Pierce™ BCA Protein Assay Kit (Thermo Fisher Scientific, Waltham, MA, USA) was used according to the manufacturer’s instructions. Equal amounts of protein samples were separated by 10% sodium dodecyl sulfate-polyacrylamide gel electrophoresis (SDS-PAGE) and transferred onto polyvinylidene difluoride membranes (GE Healthcare, Chicago, IL, USA). The membranes were blocked in 5% skim milk (Difco Laboratories, Franklin Lakes, NJ, USA) in 1x Tris-buffered saline with Tween 20 (TBST, Sigma-Aldrich) for 1 h and then incubated at 4 °C overnight with primary antibodies. The membranes were washed 3–4 times with 1x TBST for 5–10 min, followed by the incubation with the horseradish peroxidase-conjugated secondary antibodies for 45 min at room temperature. All the antibodies were diluted in 1% skim milk in 1x TBST at a ratio of 1:1000–2000. After washing 3–4 times, the membranes were imaged with ChemiDoc™ Imaging Systems (Bio-Rad Laboratories, Hercules, CA, USA) using ECL Plus Western blotting detection reagents (Amersham Bioscience, Buckinghamshire, UK). The acquired data were quantified using Image Lab™ Software (Bio-Rad).

### 4.6. Cell Migration and Invasion Assay

The Cultrex^TM^ Cell Migration Assay kit and Cultrex^TM^ Basement Membrane Extract Cell Invasion Assay kit were purchased from R&D Systems (Minneapolis, MN, USA). In the case of the cell migration assay, 50 μL of BME was added to each insert and incubated overnight in the incubator prior to cell seeding. When they reached 70–80% confluency, the cells (A549, H460, or HUVEC) were harvested and resuspended in serum-free media to be seeded into the upper chamber of the 8 μm Transwell insert at a density of 5 × 10^4^ cells/well. To the lower chamber, a serum-containing medium was added as an attractant. The plates were incubated at 37 °C for 24 h. The media in the upper chambers were then aspirated, and the bottom chambers were washed carefully using 1x Wash Buffer. Cells were incubated for 1 h with 1× Cell Dissociation Solution containing Calcein AM. The fluorescence from the detached cells was measured by an Infinite F200 Pro multimode microplate reader (Tecan, Männedorf, Switzerland) at 485 nm excitation and 520 nm emission. Relative cell migration and invasion were calculated in comparison to the controls.

### 4.7. Tube Formation Assay

The HUVECs under passage five were cultured for the tube formation assay to reach 80% confluency. Prior to the seeding, a 96-well plate was coated with 50 μL of Matrigel and incubated for complete solidification. The cells were detached and resuspended using endothelial basal medium (EBM) to reach a density of 1.5 × 10^5^ cells/mL. Equal volumes of cells were separated in microtubes and pelleted. The pellets were resuspended with EBM, 20 ng/mL VEGF, or 20 ng/mL VEGF with 10 μM AC-P19M, and 100 μL of cell suspension was added to each Matrigel-coated well. Negative control was prepared without Matrigel coating. The tube formation was observed after incubating the cells for 12 h in a cell culture incubator, and the cells were imaged under 40× magnification using an inverted light microscope.

### 4.8. Statistical Analysis

All experiments were conducted in triplicate, and the results were expressed as the mean ± standard error of the mean (SEM). The statistical significance of the data was evaluated by the one-way ANOVA test followed by Tukey’s post-test using GraphPad Prism 9.3.1 (GraphPad Software, La Jolla, CA, USA). *p*-values smaller than 0.05 were considered to indicate statistically significant differences.

## Data Availability

Not applicable.

## Supplementary Materials

The supporting information can be downloaded at: https://www.mdpi.com/article/10.3390/ijms232415594/s1.
