# Supplementary material for: De Novo Design of AC-P19M, a Novel Anticancer Peptide with Apoptotic Effects on Lung Cancer Cells and Anti-Angiogenic Activity
Source: Int J Mol Sci. 2022 Dec 9;23(24):15594. doi: 10.3390/ijms232415594 (PMC9779372; doi:10.3390/ijms232415594)

Supplementary materials

De novo design of AC-P19M, a novel anticancer peptide, with apoptotic effect on lung cancer cells and anti-angiogenic activity

Table S1. Six template sequences aligned from 15-mer to 20-mer anticancer peptides

Table S2. Representative results of functional prediction by machine learning-based web tools

Table S3. Minimum inhibitory concentration and minimum bactericidal concentration of AC-P19 and AC-P19M

Figure S1. Three-dimensional modeling of AC-P19 and AC-P19M

Figure S2. Cell cycle assay of A549 and H460 upon AC-P19M treatment

Figure S3. Field emission-scanning electron imaging of bacteria upon AC-P19M treatment

Table S1. Six template sequences aligned from 15-mer to 20-mer anticancer peptides

Peptide		Aligned sequence
15-mer	FAKKKIKGKIIKKVL	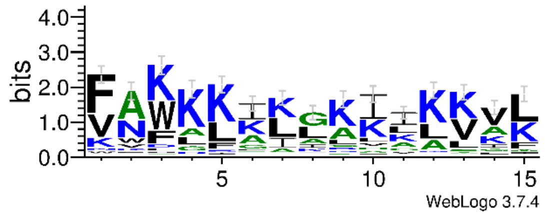
16-mer	GLFDVIKKVASVIKKL	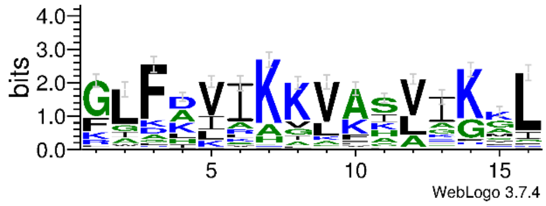
17-mer	FLKKIVKKIKKHIKKAL	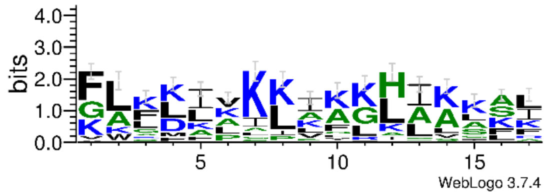
18-mer	PAWRKAKRWAWRMLKKAA	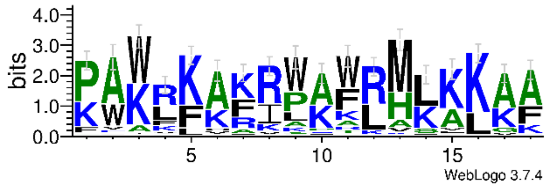
19-mer (AC-P19)	FAKKLAKLKKKLAKLALAL	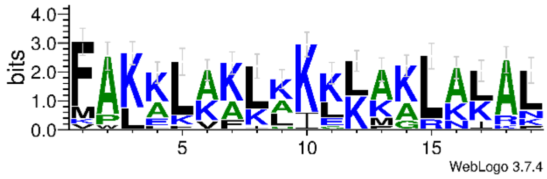
20-mer	KWKLFKKIGIGLTLPPAKAY	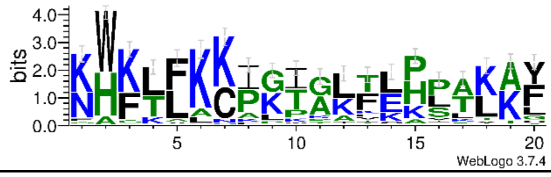

Table S2. Representative results of functional prediction by machine learning-based web tools

Length (AA)	Sequence	Anticancer activity			Cell penetrating activity			Hemolysis		
		mACPpred	ACPred	ENNACT	CellPPD	BChemRF-CPPred	MLCPP	HAPPENN	DBAASP (erythrocyte)	HemoPI
15	FAKKKIKGKIIKKVL[*]	0.9768	1	1.000	0.05 (CPP)	87.05%	0.8758	0.012	0.76 (PPV)	0.47
	FAKKKIKGKIIKKVR	0.9603	1	1.0	0.05 (CPP)	89.3%	0.9082	0.006	0.85 (PPV)	0.48
	FAKKKIKGKIIKKVLR	0.9796	1	1.0	0.05 (CPP)	89.37%	0.9186	0.012	0.85 (PPV)	0.47
16	GLFDVIKKVASVIKKL	0.9827	1	1.000	0.05 (CPP)	70.16%	0.4522	0.490	0.62 (NPV)	0.64
	GLFDVIKKVASVIKKR	0.9825	0.999	0.999	0.05 (CPP)	66.09%	0.5424	0.115	0.77 (PPV)	0.58
	GLFDVIKKVASVIKKLR	0.9797	0.998	1.0	0.05 (CPP)	62.62%	0.5225	0.302	0.50 (PPV)	0.56
17	FLKKIVKKIKKHIKKAL	0.9781	1	1.000	0.05 (CPP)	53.82%	0.9167	0.176	0.71 (PPV)	0.47
	FLKKIVKKIKKHIKKAR	0.9812	1	1.0	0.05 (CPP)	55.76%	0.9412	0.103	0.82 (PPV)	0.47
	FLKKIVKKIKKHIKKALR	0.9801	1	1.0	0.05 (CPP)	57.08%	0.9416	0.183	0.73 (PPV)	0.45
18	PAWRKAKRWAWRMLKKAA	0.9786	0.973	1.000	0.05 (CPP)	92.01%	0.9634	0.014	0.83 (PPV)	0.49
	PAWRKAKRWAWRMLKKAR	0.9787	0.912	1.0	0.05 (CPP)	93.61%	0.9795	0.008	0.96 (PPV)	0.49
	PAWRKAKRWAWRMLKKAAR	0.9795	0.961	1.0	0.05 (CPP)	93.3%	0.9757	0.008	0.88 (PPV)	0.49
19	FAKKLAKLKKKLAKLALAL[**]	0.9813	0.992	1.000	0.05 (CPP)	88.47%	0.9797	0.020	0.55 (NPV)	0.62
	FAKKLAKLKKKLAKLALAR	0.9829	0.986	1.0	0.05 (CPP)	90.6%	0.9752	0.009	0.70 (PPV)	0.62
	FAKKLAKLAKKLAKLAKKR[***]	0.9806	0.999	1.0	0.05 (CPP)	92.32%	0.9808	0.008	0.84 (PPV)	0.57

20	KWKLFKKIGIGLTLPPAKAY	0.9786	0.986	0.159	0.05 (CPP)	68.97%	0.6384	0.004	0.68 (PPV)	0.48
	KWKLFKKIGIGLTLPPAKAR	0.9781	0.908	0.251	0.05 (CPP)	63.91%	0.7379	0.002	0.85 (PPV)	0.47
	KWKLFKKIGIGLTLPPAKAYR	0.9795	0.969	0.362	0.05 (CPP)	64.03%	0.7292	0.003	0.68 (PPV)	0.50

* Underlined sequences represent aligned template sequences
** AC-P19
*** AC-P19M

Table S3. Minimum inhibitory concentration and minimum bactericidal concentration of AC-P19 and AC-P19M

**Peptide**	*Escherichia coli*		*Pseudomonas aeruginosa*		*Bacillus subtilis*	
	MIC*	MBC**	MIC	MBC	MIC	MBC
AC-P19	1	2	4	8	2	2
AC-P19M	4	32	1	2	1	1

* Minimum inhibitory concentration (μM)
** Minimum bactericidal concentration (μM)

Figure S1. Three-dimensional modeling of AC-P19 and AC-P19M

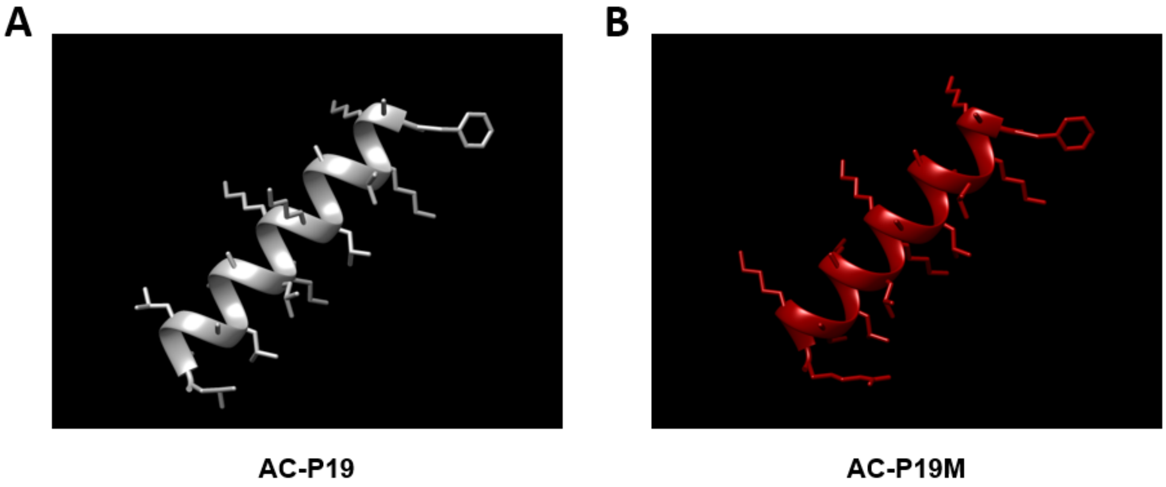

Figure S2. Cell cycle assay of A549 and H460 upon AC-P19M treatment

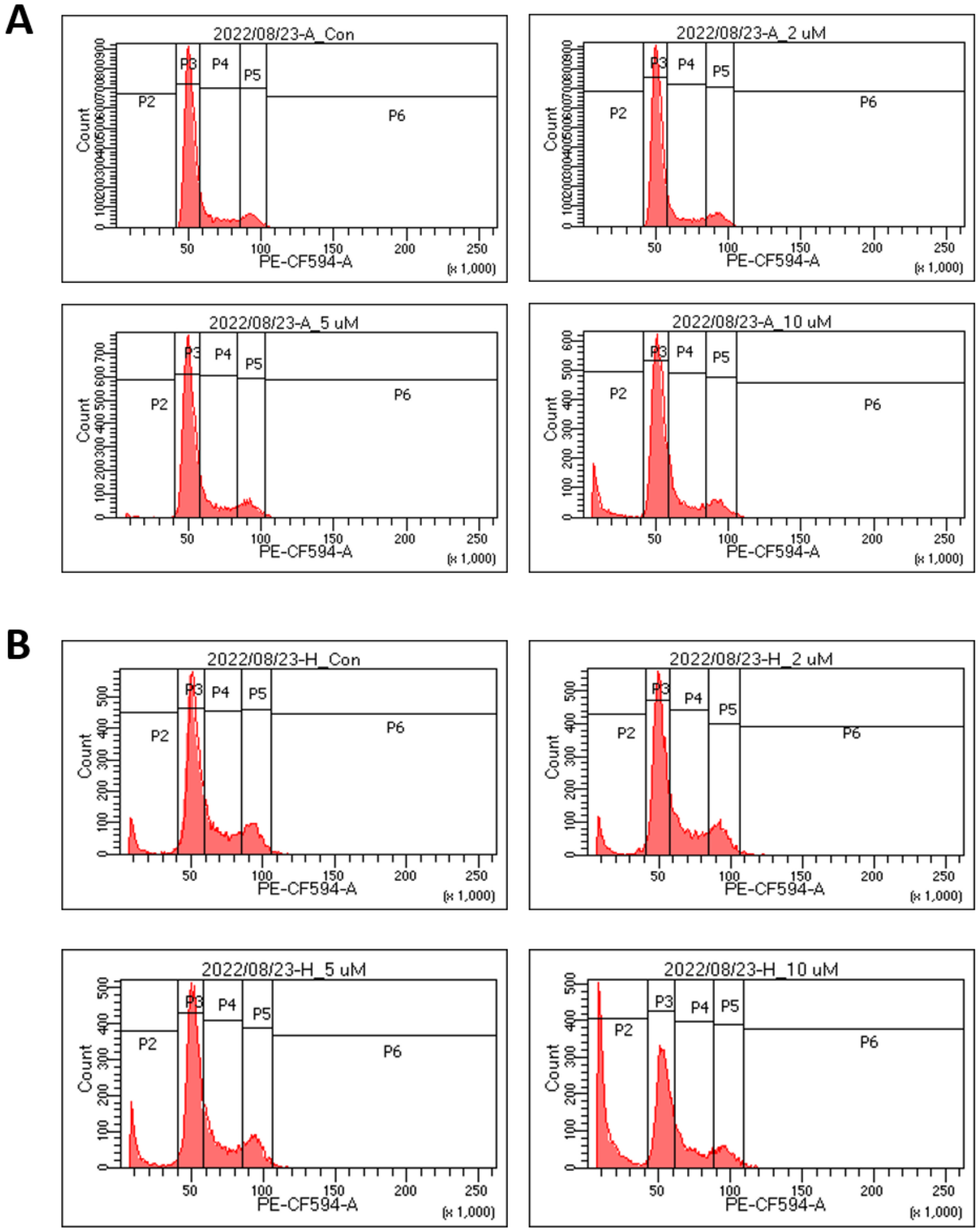

Figure S3. Field emission-scanning electron imaging of bacteria upon AC-P19M treatment

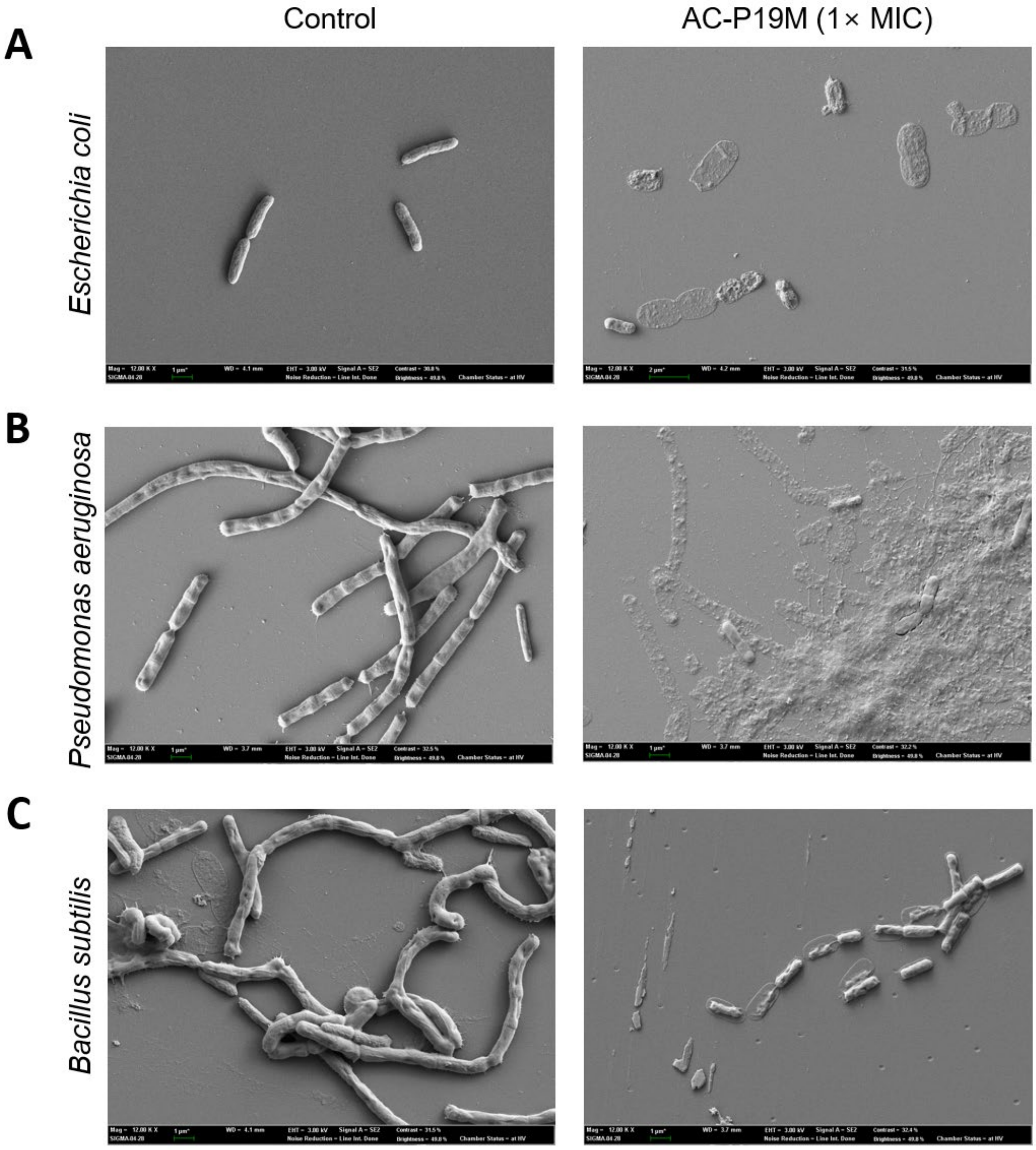

Supplement: Supplementary file 1 [file ijms-23-15594-s001.zip › ijms-2045457-supplementary.pdf]
